# Cardiac retinoic acid levels decline in heart failure

**DOI:** 10.1172/jci.insight.137593

**Published:** 2021-04-22

**Authors:** Ni Yang, Lauren E. Parker, Jianshi Yu, Jace W. Jones, Ting Liu, Kyriakos N. Papanicolaou, C. Conover Talbot, Kenneth B. Margulies, Brian O’Rourke, Maureen A. Kane, D. Brian Foster

**Affiliations:** 1 Division of Cardiology, Johns Hopkins University School of Medicine, Baltimore, Maryland, USA.; 2 Mass Spectrometry Center and Department of Pharmaceutical Sciences, University of Maryland School of Pharmacy, Baltimore, Maryland, USA.; 3 Institute for Basic Biomedical Sciences, Johns Hopkins University School of Medicine, Baltimore, Maryland, USA.; 4 Penn Cardiovascular Institute, Perelman School of Medicine, University of Pennsylvania, Philadelphia, Pennsylvania, USA.

**Keywords:** Cardiology, Heart failure

## Abstract

Although low circulating levels of the vitamin A metabolite, all-*trans* retinoic acid (ATRA), are associated with increased risk of cardiovascular events and all-cause mortality, few studies have addressed whether cardiac retinoid levels are altered in the failing heart. Here, we showed that proteomic analyses of human and guinea pig heart failure (HF) were consistent with a decline in resident cardiac ATRA. Quantitation of the retinoids in ventricular myocardium by mass spectrometry revealed 32% and 39% ATRA decreases in guinea pig HF and in patients with idiopathic dilated cardiomyopathy (IDCM), respectively, despite ample reserves of cardiac vitamin A. ATRA (2 mg/kg/d) was sufficient to mitigate cardiac remodeling and prevent functional decline in guinea pig HF. Although cardiac ATRA declined in guinea pig HF and human IDCM, levels of certain retinoid metabolic enzymes diverged. Specifically, high expression of the ATRA-catabolizing enzyme, CYP26A1, in human IDCM could dampen prospects for an ATRA-based therapy. Pertinently, a pan-CYP26 inhibitor, talarozole, blunted the impact of phenylephrine on ATRA decline and hypertrophy in neonatal rat ventricular myocytes. Taken together, we submit that low cardiac ATRA attenuates the expression of critical ATRA-dependent gene programs in HF and that strategies to normalize ATRA metabolism, like CYP26 inhibition, may have therapeutic potential.

## Graphical abstract

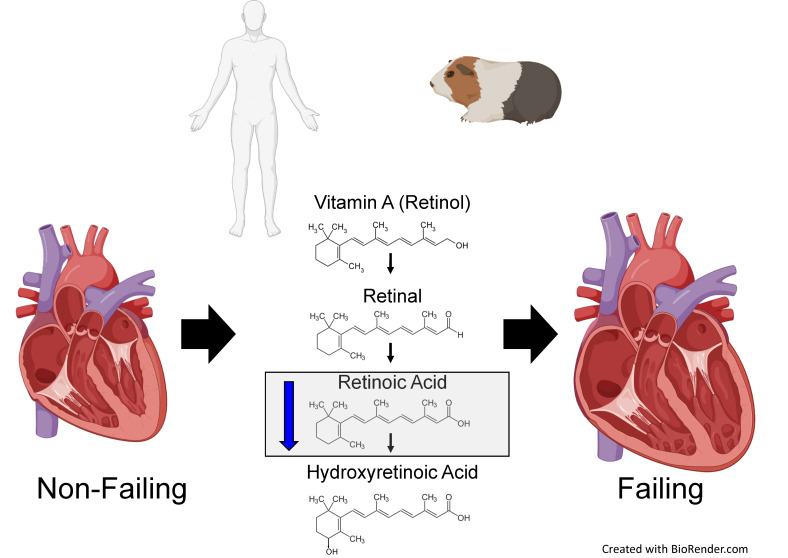

## Introduction

Vitamin A (retinol) has received clinical scrutiny in the context of coronary heart disease because low retinol correlates with increased risk of cardiac events in otherwise healthy middle-aged men (1). Like its vegetal precursor, β-carotene, whose serum levels are also inversely correlated with adverse outcomes (2, 3), retinol has antioxidant properties (4, 5), similar to vitamins E and C. Retinol has therefore been included in clinical trials, often in combination with β-carotene and/or vitamins E and C. Ultimately, antioxidant trials with or without retinol failed to improve patient outcomes (6–10).

However, unlike vitamins E and C, the major cell physiological role of retinol has little to do with direct antioxidant action. Stored primarily in the liver, retinol is a prohormone, the body’s source of all-*trans* retinoic acid (ATRA), a potent transcription-activating hormone present in tissues at nanomolar concentrations. Over 70 years ago, it was first shown that the offspring of retinol-deficient rats exhibited structural cardiac defects (11, 12). The role of ATRA in embryonic cardiac development has subsequently been mapped in considerable detail. It is involved in proper cardiac tissue specification, anteroposterior patterning, and endocardial cushion formation, among other processes (13–16). Yet, the cellular role of the retinoids, their metabolism, and signaling in adult heart physiology and heart failure (HF) remain poorly characterized. There are no interventional trials that have evaluated the impact of vitamin A or ATRA alone on HF (17) and observational clinical trials have not shown any difference in serum retinol levels between HF patients and sex-matched healthy controls (18). Although HF patients do not appear to be retinol deficient, it is unknown whether ATRA levels are altered. Indeed, the precise relationships between circulating retinol and ATRA levels, cardiac tissue ATRA levels, and intracellular cardiac ATRA signaling in the context of HF are unknown.

Only a few studies have examined the mechanism by which altered ATRA signaling might affect the function of the adult mammalian heart. Specifically, tamoxifen-inducible cardiac-specific deletion of one of ATRA’s cellular targets, the retinoic acid receptor α (RAR-α) is sufficient to elicit diastolic dysfunction without affecting left ventricular ejection fraction (LVEF) (19). At the cellular level, RAR-α deletion exacerbates myocardial oxidative stress and blunts SERCA-mediated sarcoplasmic reticulum Ca^2+^-reuptake. Given that curbing ATRA signaling causes cardiac dysfunction in the adult mouse heart, here we have sought to understand whether an ATRA signaling defect exists in human and animal models of HF.

ATRA signaling activity in failing human myocardium was assessed by direct quantification of retinol, ATRA, and other endogenous retinoid metabolites. We show that myocardial ATRA declined in both human and experimental HF, despite ample local retinol reserves. The decline arose early in HF progression in guinea pigs, and forestalling it prevented HF. Interestingly, despite sharing low ATRA levels, the expression of ATRA-metabolizing enzymes differ between human and guinea pig HF, which could have implications for therapeutic strategy.

## Results

### Coordinate protein downregulation in the human HF proteome is consistent with decreased cardiac ATRA.

To gauge the prospects for a retinoid signaling defect, we performed a de novo analysis of a recently published high-quality proteomic data set (20). The study encompassed patients with HF arising from several pathologically or etiologically distinct forms of HF, including HF with reduced ejection fraction (HFrEF, *n* = 5), HF with preserved ejection fraction (HFpEF, *n* = 4), ischemic cardiomyopathy (ICM, *n* = 6), and idiopathic dilated cardiomyopathy (IDCM, *n* = 6). Nonfailing hearts (LVEF > 50%) fell into 2 groups, those with hypertrophy (cHYP, *n* = 6) and those without (Normal, *n* = 7).

**Figure 1:**
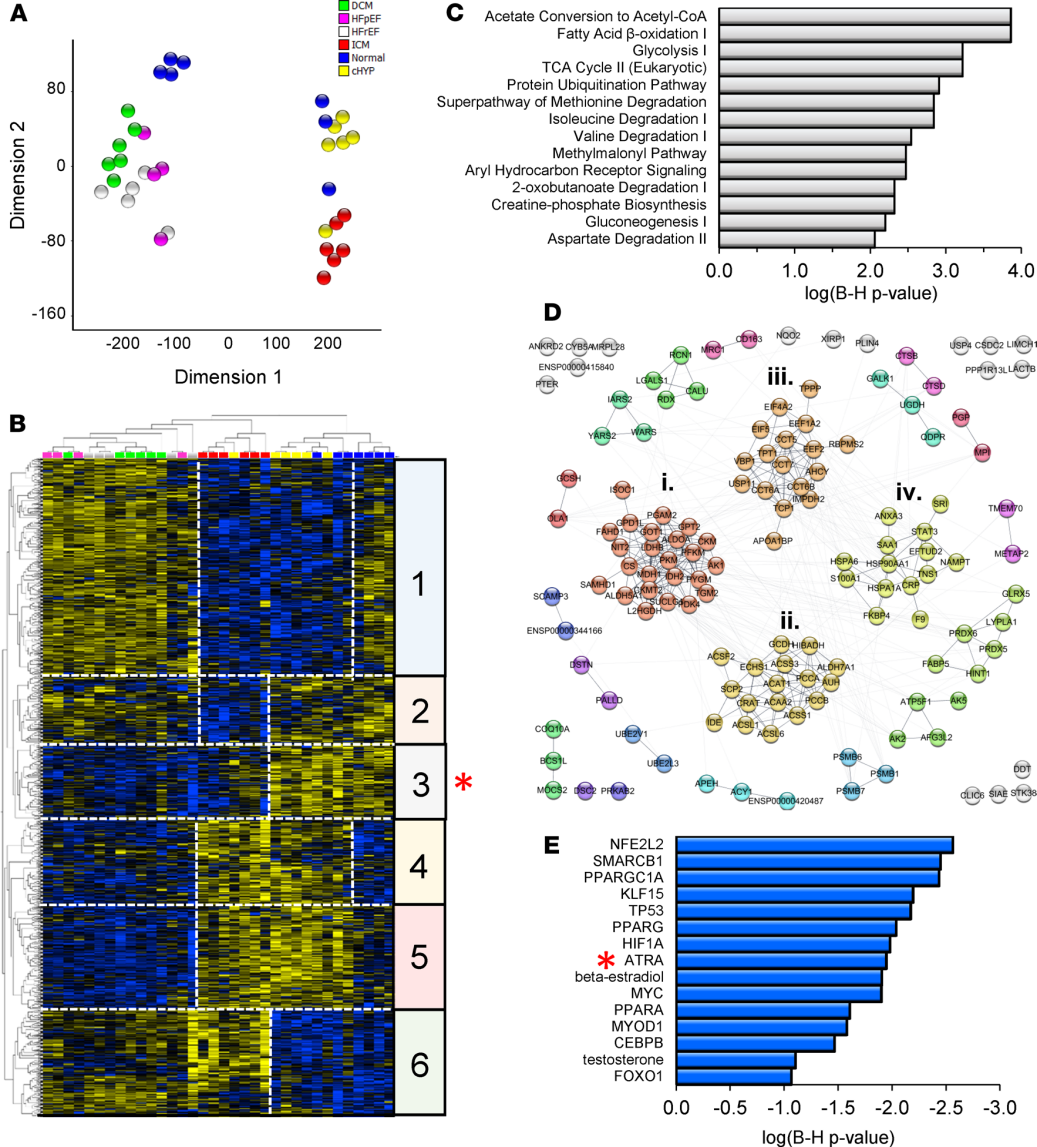
Human cardiac proteomes are consistent with a decline of the vitamin A metabolite and transcriptionally active hormone, ATRA, across human heart failure etiologies. (**A**) Dimension reduction (t-SNE) of significantly regulated proteins across 4 HF etiologies and compensated hypertrophy (LIMMA, *P* < 0.05). Normal, myocardium from healthy donors (blue; *n* = 7); cHYP, compensated hypertrophy (yellow; *n* = 6); HFrEF, HF with reduced ejection fraction (white; *n* = 5); HFpEF, HF with preserved ejection fraction (pink; *n* = 4); IDCM, idiopathic dilated cardiomyopathy (green; *n* = 6); ICM, ischemic cardiomyopathy (red; *n* = 6). HFrEF, HFpEF, and IDCM proteomes share substantial similarity, whereas ICM has a distinctive biosignature. (**B**) Hierarchical clustering of significantly regulated proteins (blue, downregulated; yellow, upregulated). HF samples follow the color scheme from **A**. Protein levels largely correlate across HFpEF, HFrEF, and IDCM (e.g., clusters 1, 4, and 5). Cluster 2 depicts proteins uniquely downregulated in ICM. Clusters 3 and 6 represent proteins similarly regulated across HF etiologies. Specifically, cluster 3 (red asterisk) represents 132 proteins that are downregulated in most HF patients. (**C**) Pathway analysis showed that these proteins fall into pathways widely viewed as metabolic hallmarks of HF. (**D**) Coordinately downregulated proteins constitute a bona fide multimodular protein association network. (**E**) Upstream regulator analysis to identify transcriptional programs that might explain coordinate downregulation and activity of the network. ATRA (red asterisk) activity is inferred to decrease.

Empirical-Bayesian statistical analysis (LIMMA) was performed to identify differentially regulated proteins in the data set. Trends and similarities among significantly regulated proteins (1144; *P* < 0.05) are summarized by dimension reduction (t-distributed stochastic neighbor embedding, t-SNE; Figure 1A) and hierarchical clustering (Figure 1B). HFrEF, HFpEF, and IDCM proteomes shared substantially similar protein profiles at this depth of coverage (Figure 1B, clusters 1, 4, 5, and 6). HF secondary to ischemia exhibited a distinct proteome (Figure 1B, cluster 2), differing from other forms of HF, as well as the proteomes of patients with nonfailing hypertrophic hearts (cHYP) and nonfailing nonhypertrophic hearts (Normal). Nevertheless, hierarchical clustering showed a subset of differentially downregulated proteins common to both ischemic and nonischemic HF (Figure 1B, cluster 3, 135 proteins, red asterisk). These proteins are associated with processes now broadly understood to be impaired in human HF, including fatty acid β-oxidation, branched-chain amino acid (BCAA) metabolism, the Krebs cycle, and creatine phosphate metabolism (Figure 1C). These processes are interconnected in a bona fide functional network (Figure 1D) consisting of 136 nodes connected by 455 edges (connections), which is substantially greater than would be expected by chance (139 edges, *P* < 1 × 10^–16^). Markov clustering revealed 12 functional modules (protein nodes with high connectivity) containing at least 3 proteins. The 4 largest modules within cluster 3 are highlighted in Figure 1D. They encompass (i) glycolysis/TCA cycle/creatine phosphate biosynthesis, (ii) fatty acid oxidation/BCAA metabolism, (iii) protein synthesis/TCP1 ring complex, and (iv) other chaperones and Ca^2+^-binding proteins. To gain insight into potential transcriptional programs that might be consistent with coordinate protein downregulation within this cluster, we performed upstream regulator analysis across HF types (Ingenuity Pathway Analysis, QIAGEN; Figure 1E). Shown are statistically significant transcriptional regulators (*P* ≤ 0.05, Fisher’s exact test, both proteins and metabolites) ranked by their *z* scores or level of inferred inhibition. Among transcriptionally active metabolites, the action of ATRA was inferred to be the most inhibited (*z* score= –1.95, *P* = 0.05, red asterisk). Indeed, an ATRA deficit would be directly consistent with coordinate downregulation of at least 10 other proteins in cluster 3. Moreover, ATRA has demonstrated relationships with 9 other transcription factors implicated in Figure 1E (see Supplemental Figure 1; supplemental material available online with this article; https://doi.org/10.1172/jci.insight.137593DS1). Therefore, coordinate downregulation of cardiac proteins in human HF is consistent with low levels of ATRA.

### Mass spectrometry shows ATRA decline in the failing human heart.

Levels of ATRA and other endogenous retinoids were assessed directly in human HF myocardium using HPLC-UV and LC-multistage tandem mass spectrometry (LC-MRM^3^) in a blinded manner. The patient characteristics of the 20-patient cohort used in the study are summarized in Table 1. The cohort consisted of men (*n* = 9) and women (*n* = 11), including 13 White patients, 4 African American patients, and 2 patients of unknown race. Neither age nor BMI differed significantly between nonfailing and failing (IDCM) groups. Nonfailing hearts (*n* = 10) weighed 368 ± 74 g, whereas failing hearts (IDCM; *n* = 10) that had undergone chamber dilation and enlargement were 62% heavier (596 ± 88 g; *P* < 0.00001). Nonfailing hearts had an average LVEF of 53% ± 3% compared with 15% ± 4% (*P* < 0.0001) for failing hearts.

**Figure 2:**
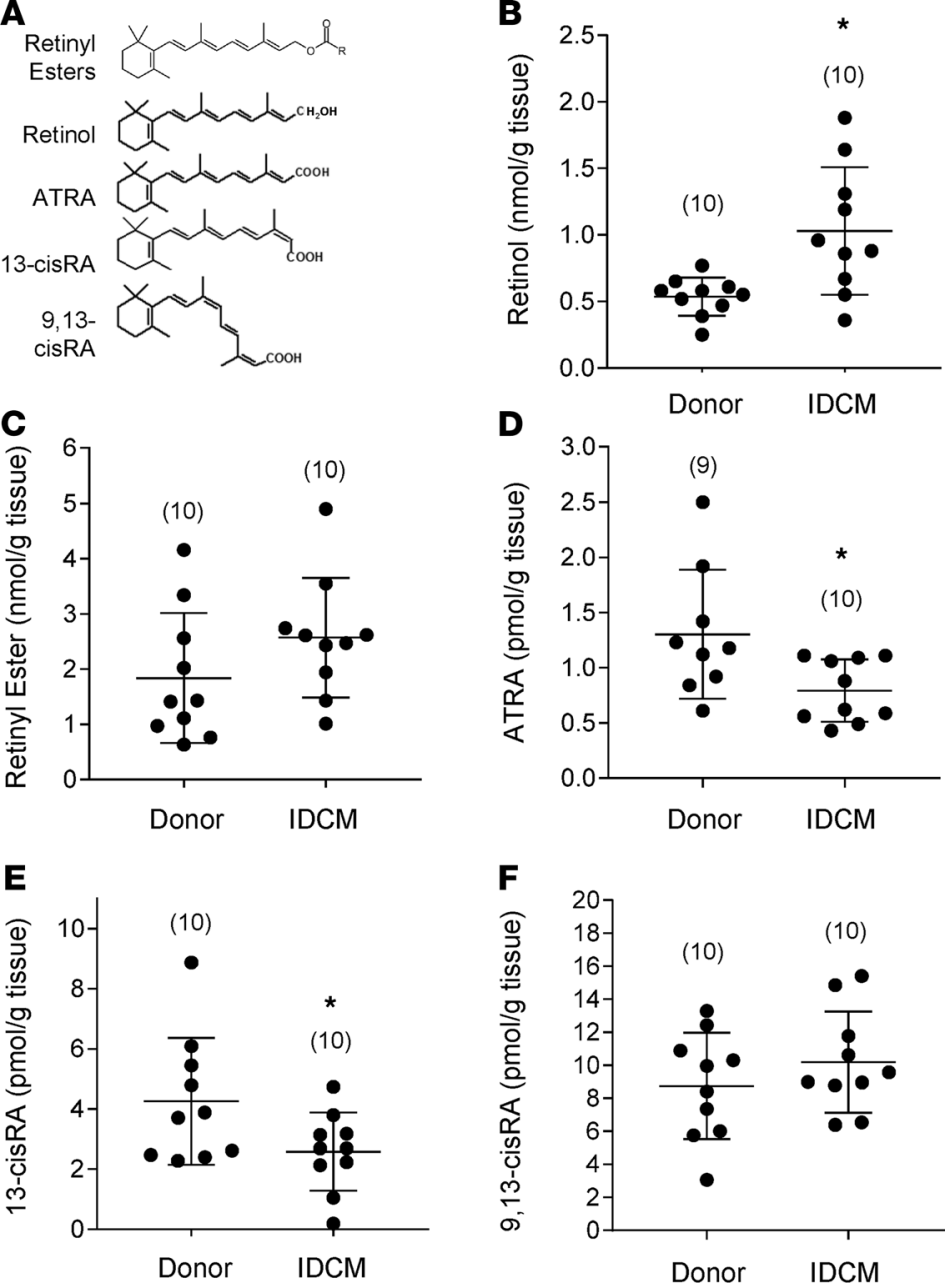
ATRA but not vitamin A declines in idiopathic dilated cardiomyopathy. (**A**) Chemical structures of the retinoid species quantified from myocardial biopsies by reversed-phase chromatography and mass spectrometry. Retinyl esters represent the in situ retinoid reserve, or storage form, of retinol. Retinol, or vitamin A, is the metabolic precursor of retinaldehyde and all-*trans* retinoic acid (ATRA). Other geometric isomers of retinoic acid include 13-*cis*-RA and 9,13-*cis*-RA. The potent RXR ligand, 9-*cis*-RA, was not detected in the heart. (**B**–**F**) Quantitation of endogenous cardiac retinoids. Differences between group means were assessed by a 2-tailed *t* test (* denotes *P* < 0.05). *N* values are given in parentheses. (**B**) Retinol levels were significantly elevated in patients with idiopathic dilated cardiomyopathy (IDCM) relative to donor heart tissue. (**C**) A similar trend was noted for retinyl esters, though the difference was not significant. (**D**) ATRA was significantly lower in IDCM than among donors, as were levels of 13-*cis*-RA (**E**). (**F**) 9,13-*cis*-RA did not differ significantly.

In Figure 2A, we show the chemical structures of the retinoid species quantified from human cardiac tissue, which include retinyl esters (the cellular storage form retinol), retinol, ATRA, and other endogenous isomers 13-*cis*-RA and 9,13-*cis*-RA, whose cellular roles are still not clearly defined. The potent retinoid X receptor (RXR) agonist, 9-*cis* RA, was not detectable in human hearts despite a detection limit for the mass spectrometry assay at the femtomole level (<0.05 pmol/g tissue). Levels of retinol in human myocardium were significantly higher in the IDCM patient group (1.03 ± 0.47 [*n* = 10] vs. 0.54 ± 0.14 nmol/g tissue [*n* = 10], *P* < 0.05). Levels of retinyl esters perhaps trended higher, though failed to reach significance (1.84 ± 1.18 [*n* = 10] vs. 2.57 ± 1.08 nmol/g [*n* = 10], *P* = 0.16). Notably, however, ATRA was 39% lower in the myocardium of IDCM patients relative to donor controls (0.79 ± 0.28 [*n* = 10] vs. 1.30 ± 0.58 pmol/g [*n* = 9], *P* < 0.05). The decline in ATRA coupled with increased retinol is consistent with an impairment in ATRA biosynthesis. The ATRA decline was mirrored in levels of the 13-*cis*-RA isomer, which also fell 39% in IDCM (4.26 ± 2.11 [*n* = 10] vs. 2.58 ± 1.30 pmol/g [*n* = 10], *P* < 0.05). Levels of 9,13-*cis*-RA were unchanged between groups. Multifactor ANOVA was used to verify that significant differences in ATRA levels were not due to the potentially confounding effect of sex or race (Supplemental Figure 2). HF diagnosis was the only significant factor contributing to variance in ATRA levels (*P* = 0.019).

### Coordinate protein downregulation across the stages of guinea pig HF implicates retinoid pathways.

**Figure 3:**
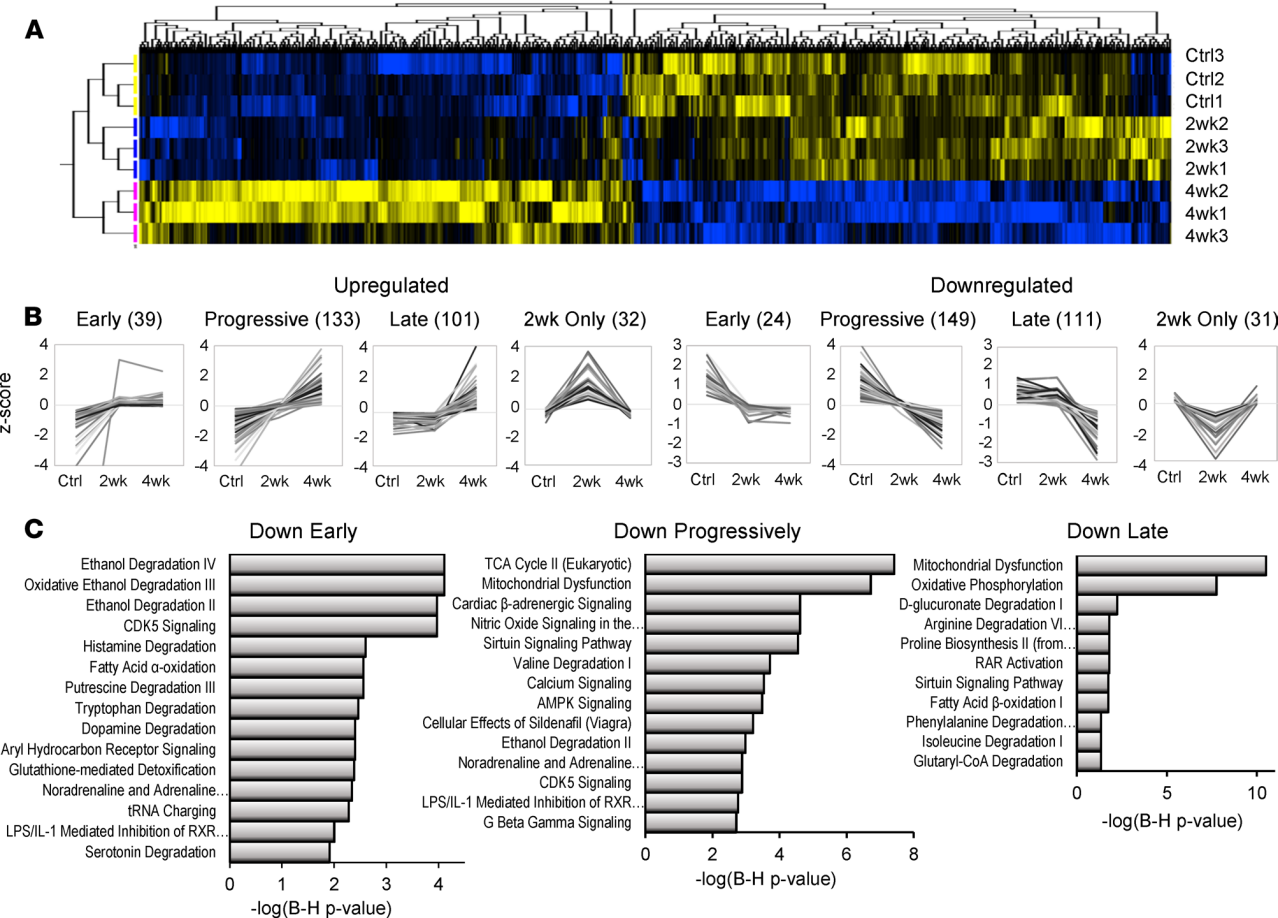
Trajectories of proteomic changes in guinea pig heart failure progression implicate retinoid pathways from early to late stages. (**A**) Hierarchically clustered heatmap of differentially regulated proteins in guinea pig HF progression (LIMMA, *P* < 0.05). Groups include the control (Ctrl; *n* = 3), 2 weeks of ACi protocol (2wk; *n* = 3), and 4 weeks of the ACi protocol (4wk; *n* = 3); 924 of 4150 were deemed significantly regulated. Careful inspection of the heatmap revealed 8 observable clusters corresponding to trajectories of protein expression. (**B**) Identification of proteins whose group means (*z* score normalized) most closely correlated (Pearson’s *r* > 0.975) with the 8 observed trajectories of up or downregulation. Numbers in parentheses indicate the number of proteins classified in each group; 620 of 924 proteins met the strict correlation criterion. (**C**) Pathway analysis was performed on proteins whose trajectories were classified as early, progressive, or late movers (Fisher’s exact test). After Benjamini-Hochberg correction, retinoid pathways were identified (*P* < 0.05). An RXR pathway was implicated in early and progressive trajectories. Several proteins downregulated at 4 weeks only contributed to the identification of the RAR activation pathway.

We sought to determine the extent to which the human proteomic biosignatures and retinoid profile of HF were recapitulated in an experimental model and over what timescale of HF progression. Here, we used the guinea pig ACi model (aortic constriction and daily low-dose isoproterenol) that demonstrated a phase of compensated hypertrophy (2 weeks) followed by rapid loss of pump function (decompensation) by 4 weeks. We previously published an integrated transcriptomic, proteomic, and metabolomic analysis of the guinea pig ACi model (21), though failed to identify endogenous retinoids in our initial metabolomic screen, which is not surprising given their relatively low abundance, the limited ability of global metabolomic screens to detect low abundance analytes, and the inefficiency of typical global metabolomic sample preparation methodology for the extraction of retinoids. In Figure 3, using empirical Bayesian statistics, hierarchical clustering, and correlation profiling, we were able to classify the trajectory of protein changes as a function of HF progression. Briefly, of the 4150 proteins identified in 3 independent iTRAQ proteomics experiments (*n* = 3 per group), 994 proteins were differentially regulated, clustered in Figure 3A (*P* < 0.05). Inspection of Figure 3A, as well as initial *k* means clustering, revealed that differentially regulated proteins fell into 8 principal groups, 4 upregulated and 4 downregulated. For both up- and downregulated groups, some protein levels changed within 2 weeks and remained largely unchanged thereafter (early). There were also proteins whose changes progressed linearly as a function of HF (progressive) and those whose relative levels only changed substantially between 2 and 4 weeks (late). Finally, there were a few proteins whose levels differed from controls substantially at 2 weeks only. Proteins were ranked by correlation to models of these 8 trajectories to identify proteins that best exemplified each class. Protein levels that correlated highly (Pearson’s *r* > 0.975) are depicted in Figure 3B. Briefly, the 8 classes encompassed 620 of 994 significantly regulated proteins. Progressive changes were the most common (282 proteins), followed by late protein changes (212 proteins). Comparatively few classified as early movers (63 proteins).

To understand the impact of this regulation, particularly with respect to processes shown to be downregulated in Figure 1, pathway analysis was performed on the Down Early, Down Progressively, and Down Late classes. The Down Early pathways are dominated by pathways that feature aldehyde dehydrogenase 2 (Aldh2) as a common element, including “ethanol degradation” and a retinoid signaling pathway “LPS/IL1-mediated inhibition of RXRα.” Pathways implicated in the Down Progressively class include the TCA cycle (specifically succinate dehydrogenase [SDH] subunits), mitochondrial dysfunction (SDH and ATP synthase subunits), cardiac β-adrenergic signaling (including the major channels of excitation-contraction coupling), and valine degradation. “Ethanol degradation” is again significant, owing in part to downregulation of the retinaldehyde-metabolizing enzyme, retinal dehydrogenase 1 (Aaldh1a1), which IPA attributes to this pathway. The aforementioned “LPS/IL1-mediated inhibition of RXRα” pathway is also implicated. Finally, Down Late pathways include oxidative phosphorylation and fatty acid β-oxidation and metabolism of amino acids isoleucine, proline, and arginine. Here, however, RAR activation is specifically implicated. Taken together, pathway analysis of downregulated proteins implicates retinoid-metabolizing enzymes and retinoid signaling pathways across all phases of HF progression in the guinea pig.

### Cardiac ATRA declines early in guinea pig HF.

**Figure 4:**
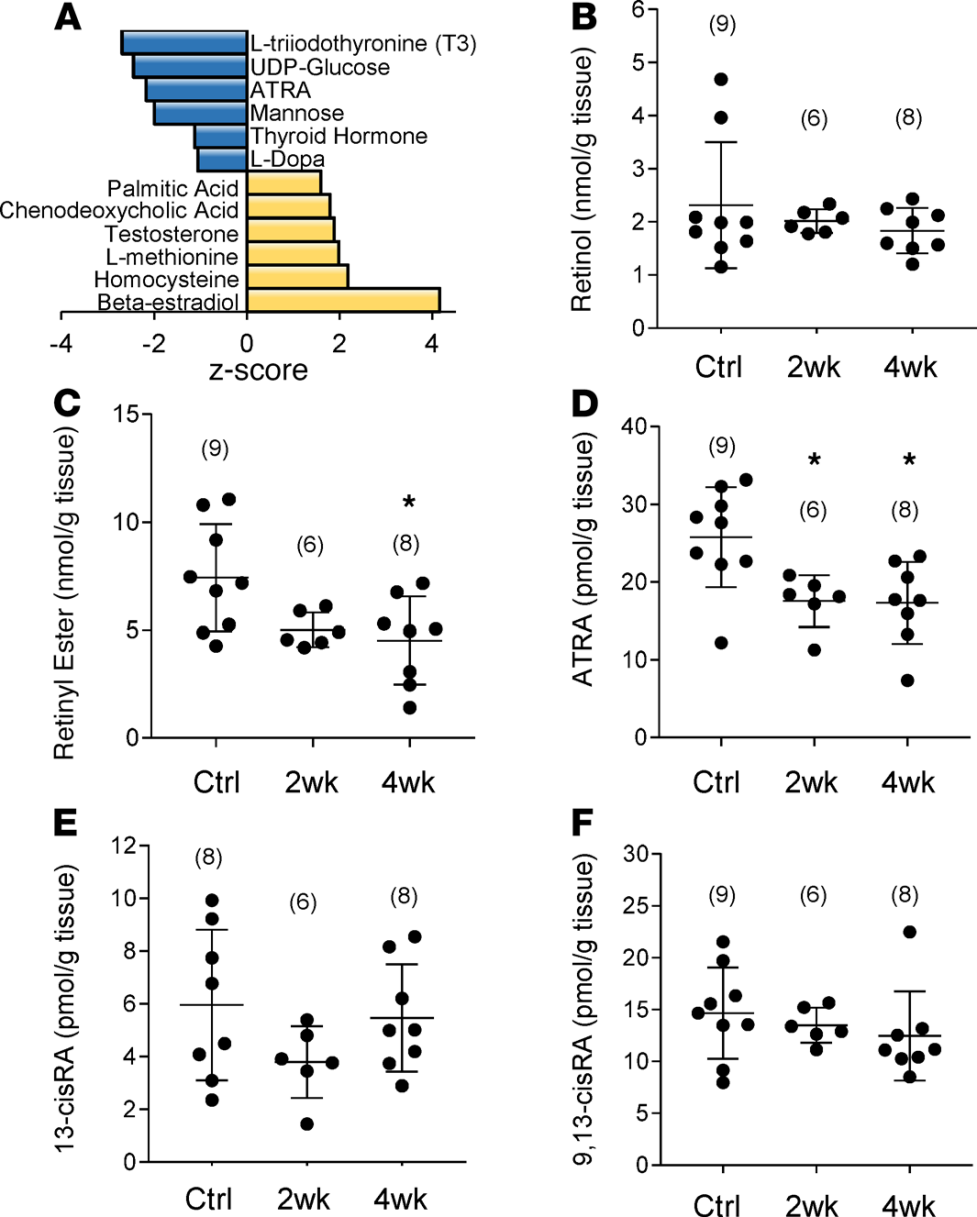
Low cardiac ATRA is recapitulated early in guinea pig heart failure progression. (**A**) URA analysis identified endogenous chemical compounds inferred to be altered, given the differential regulation of proteins in Figure 3A. Select compounds (Fisher’s exact test, *P* < 0.05, *z* score > 1.0) are shown. Low ATRA was specifically inferred from URA of guinea pig HF. **B**–**F** show the results of the analysis of cardiac retinoids. Experimental groups are as defined in Figure 3. Group means were analyzed by 1-way ANOVA with a post hoc Tukey’s HSD test and *P* < 0.05 was considered significant. *N* values for each group are given in parentheses. (**B**) Retinol levels were unchanged from controls to HYP and HF. (**C**) The storage form of retinol, retinyl esters, declined significantly in HF but was already trending lower in HYP. (**D**) ATRA was significantly decreased in both HYP and HF, whereas other geometric isomers of retinoic acid did not differ significantly (**E** and **F**). As in human myocardium, 9-*cis*-RA was not detected. *P < 0.05.

Consistent with implicated pathways, upstream regulator analysis of downregulated proteins (irrespective of class) indicated that among transcriptionally active metabolites, ATRA action was inferred to be inhibited (Figure 4A, *z* score = –2.18, *P* = 0.0003). Another implicated metabolite was L-triiodothyronine (*z* score = –2.67, *P* = 0.001), the bioactive form of thyroid hormone, whose levels were previously known to be deficient in advanced human HF (22, 23). Resident cardiac retinoids in guinea pig myocardium were quantified in a blinded manner as described in Figure 2. Specifically, guinea pig control (Ctrl), 2-week, and 4-week HF hearts were analyzed by quantitative mass spectrometry. Levels of retinol (Figure 4B), were on the order of 2 nmol/g tissue and did not differ significantly between the groups. Interestingly, retinyl ester (Figure 4C) declined by 2 weeks (5.02 nmol/g ± 0.81 [*n* = 6]), becoming significant at 4 weeks (4.52 nmol/g ± 2.03 [*n* = 8]; *P* < 0.05) relative to controls (7.44 nmol/g ± 2.48 [*n* = 9]). ATRA (Figure 4D) declined significantly by 2 weeks (32% reduction; 17.57 pmol/g ± 3.33 in HYP [*n* = 6]; *P* < 0.05) and the decrease was sustained at 4 weeks (33% reduction; 17.32 pmol/g ± 5.26 [*n* = 8]; *P* < 0.05) with respect to controls (25.80 pmol/g ± 6.45 [*n* = 9]). Neither 13-*cis*-RA nor 9,13-*cis*-RA differed between experimental groups. Finally, we note that treatment with CGP37157, an inhibitor of the mitochondrial Na/Ca exchanger that prevents guinea pig HF by preserving mitochondrial Ca^2+^ homeostasis and the mitochondrial antioxidant pool (24), also prevented the decline in ATRA in the guinea pig HF model (see Supplemental Figure 3).

### ATRA protects hearts from pressure overload–induced HF.

**Figure 5:**
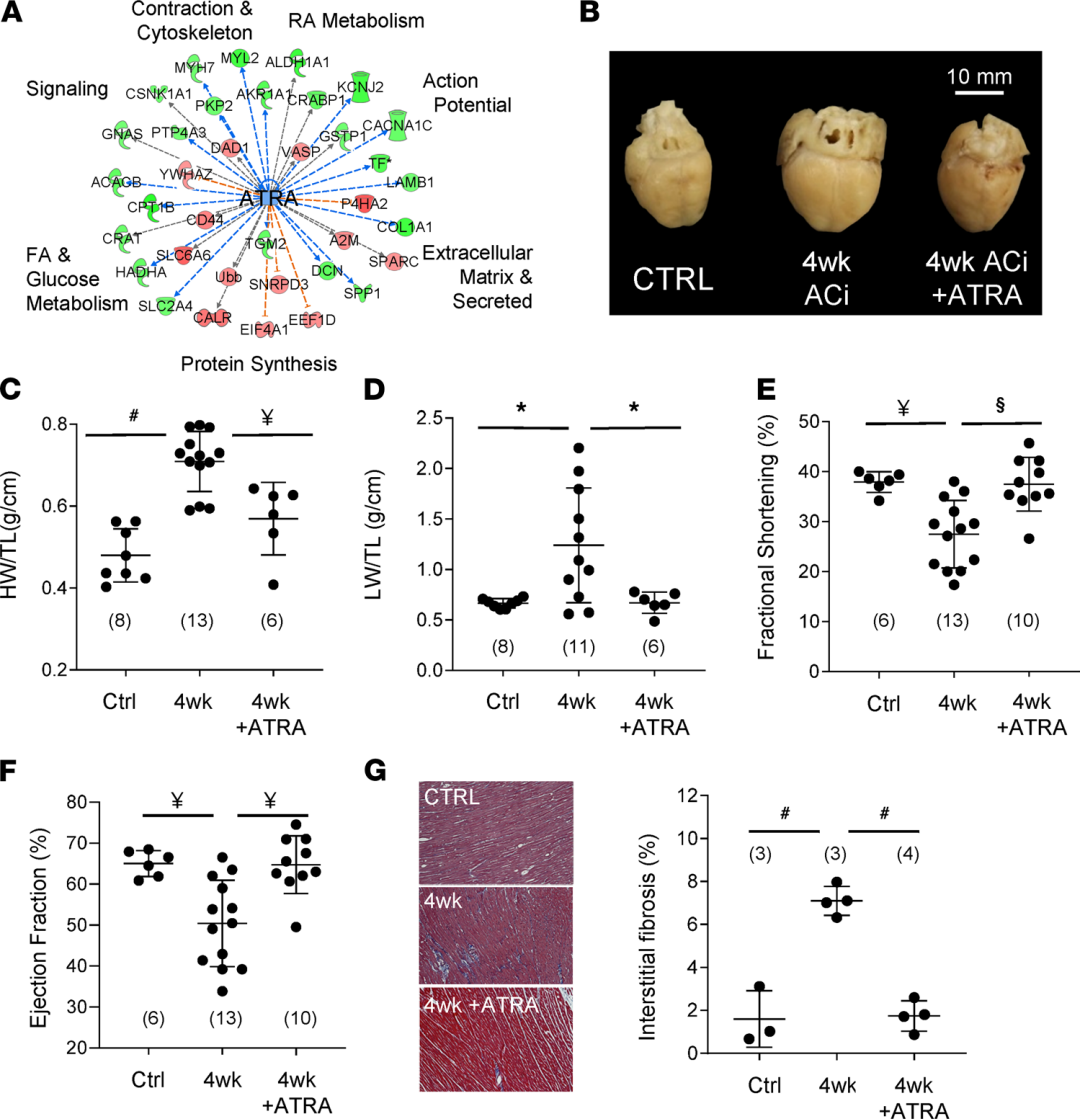
ATRA prevents experimental guinea pig heart failure. (**A**) Proteins differentially regulated in guinea pig HF (LIMMA *P* < 0.05), whose modulation by ATRA has been documented (IPA knowledge base), span hallmark processes of HF, suggesting the therapeutic potential of ATRA supplementation. (**B**) Representative hearts from sham-operated (Ctrl), 4 weeks after aortic constriction (4wk), and 4 weeks of treatment with 2 mg/kg/d of ATRA (4wk+ATRA). **C**–**G** depict structural and functional analyses of guinea pig hearts and lungs, analyzed by 1-way ANOVA with a Tukey’s post hoc HSD test. (^#^: *P* ≤ 0.0001, ^§^: *P* < 0.001, ^¥^: *P* < 0.01, *: *P* < 0.05). *N* values are given in parentheses. (**C**) ATRA prevented cardiac hypertrophy (HW: heart weight; TL: tibia length) and (**D**) pulmonary congestion (LW: lung weight). (**E**) ATRA treatment prevented declines in fractional shortening and (**F**) ejection fraction. (**G**) The impact of ATRA on accruing interstitial fibrosis was assessed. Representative images are shown on the left (10× magnification) and ensemble data on the right.

Figure 5A depicts genes altered in experimental HF that are indirectly ATRA responsive. Low ATRA levels would be consistent with proteomic changes in β-adrenergic signaling (e.g., GNAS), calcium handling (RYR2), fatty acid metabolism (CPT1B), protein synthesis (EEF1), and extracellular matrix remodeling (COL1A2), among other targets. Therefore, ATRA treatment might be expected to forestall HF in guinea pigs. In Figure 5B, representative hearts are shown from sham-operated guinea pigs (Ctrl), guinea pigs subjected to 4 weeks of the ACi protocol (4wk), and those that were subjected to 4 weeks ACi and treated with ATRA at 2 mg/kg/d (4wk + ATRA). ATRA treatment substantially limited enlargement of the heart. Ensemble data are provided in Figure 5C. Specifically, after 4 weeks, heart weights (normalized to tibia length, HW/TL) were 48% heavier (ACi: 0.71 g/cm ± 0.07 [*n* = 13]; *P* < 0.01) than the sham-operated control group (0.48 g/cm ± 0.07 [*n* = 8]), whereas hypertrophy was abrogated by ATRA treatment (0.57 g/cm ± 0.09 [*n* = 6]; *P* < 0.01). ATRA treatment also completely blunted pulmonary congestion associated with pressure overload (Figure 5D). Normalized lung weights (LW/TL) were broadly distributed in the 4-week HF group (1.24 g/cm ± 0.6 [*n* = 11]), nearly double that of the control group (0.67 g/cm ± 0.05 [*n* = 8]; *P* < 0.05). Lung weights from the ATRA-treated group were similar to controls (0.67 g/cm ± 0.11 [*n* = 6]) but differed significantly from the 4-week HF group (*P* < 0.05). Echocardiography was used to calculate fractional shortening (Figure 5E) and ejection fraction (Figure 5F). After 4 weeks, the fractional shortening of the ACi group declined by 38% (27.5% ± 6.75% [*n* = 13]; *P* < 0.01) relative to the control group (37.9% ± 2.08% [*n* = 6]). ATRA treatment, in contrast, prevented the decline (37.5% ± 5.37% (*n* = 10); *P* < 0.01). Likewise, 4 weeks of ACi protocol reduced ejection fraction from 65.0% ± 3.1% (*n* = 6; *P* < 0.01) in the control group to 50.4% ± 10.5% (*n* = 13) in ACi (Figure 5F). This, too, was prevented by concomitant treatment with ATRA (64.7% ± 7.0% [*n* = 10]; *P* < 0.01). Echocardiographic measurements are summarized in Supplemental Table 1. ATRA treatment also prevented HF-associated increases in interstitial fibrosis (Figure 5G). Specifically, the extent of fibrosis increased nearly 4.5-fold at 4 weeks of HF (7.1% ± 0.68% vs. 1.6% ± 1.32% in controls, *P* = 0.0001), whereas fibrosis in the ATRA-treated group was limited to 1.7% ± 0.70% (*P* < 0.0001 with respect to 4-week group). Finally, we confirmed that the ATRA treatment resulted in increased intracardiac ATRA (87%, *P* < 0.05) relative to the 4-week HF group (Supplemental Figure 4).

### Enzymes responsible for ATRA metabolism are regulated differently between human and guinea pig HF.

**Figure 6:**
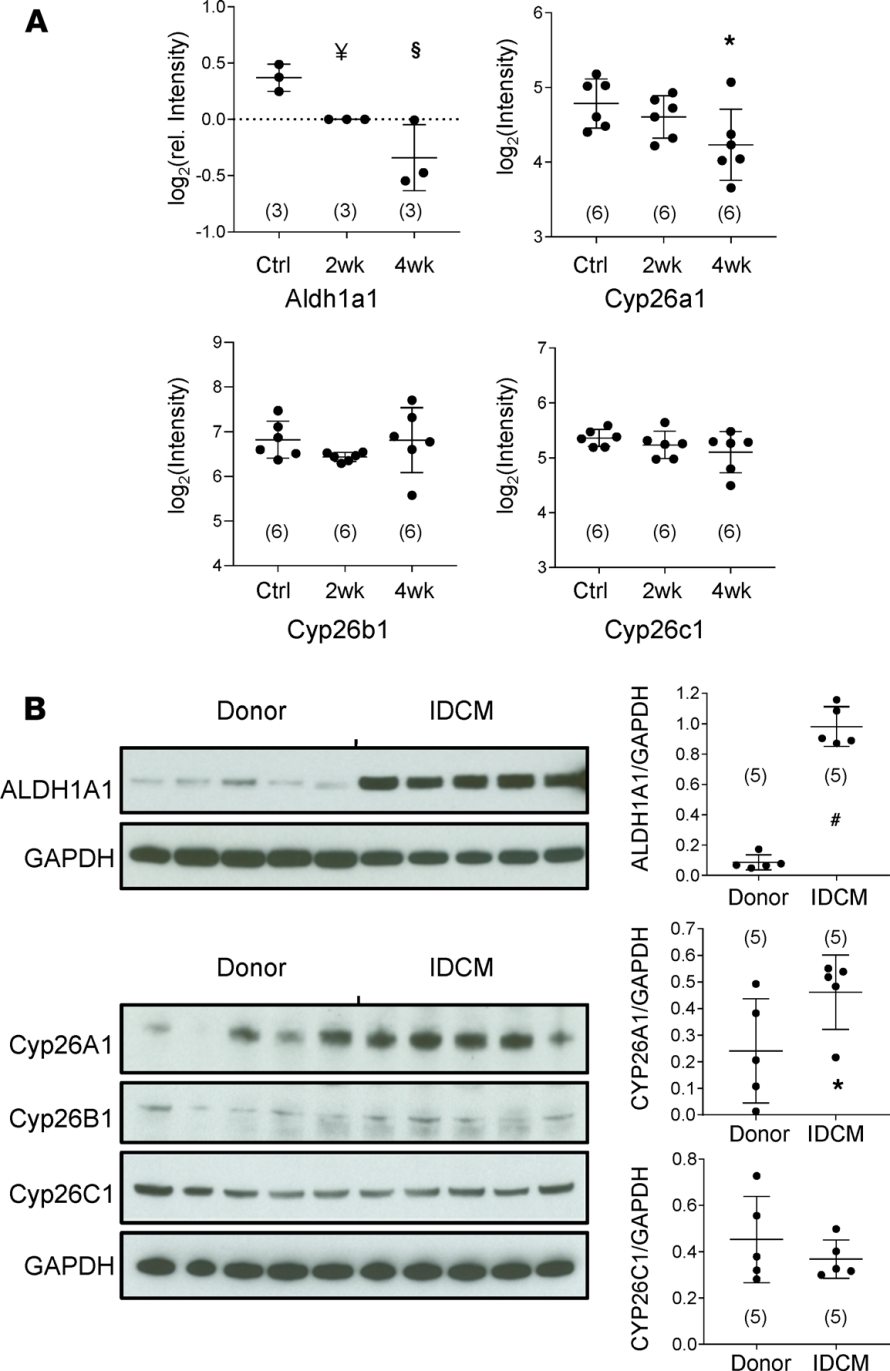
Remodeling of the enzymes of ATRA metabolism differs between guinea pig and human heart failure. (**A**) Guinea pig HF: Groups are as defined in Figure 3. *N* values are given in parentheses. ALDH1A1 protein levels followed a trend of progressive downregulation in HF that differed significantly from Ctrl at 2 weeks and 4 weeks. LIMMA-moderated *t* test as in ref. 21; ^§^: *P* < 0.001, ^¥^: *P* < 0.01. Transcript levels of CYP26A1 fell significantly by 4-week HF, whereas levels of CYP26B1 and CYP26C1 were unchanged (1-way ANOVA, post hoc Tukey’s HSD; *: *P* < 0.05). (**B**) Human IDCM: immunoblot signal intensities were analyzed as detailed in Methods. Group mean differences were assessed by a 2-tailed *t* test (^#^: *P* < 0.0001). Immunoblots indicate that retinaldehyde dehydrogenase 1 (ALDH1A1) was expressed at low levels in the myocardium of healthy donors but was increased approximately 10-fold in patients with IDCM. CYP26A1 levels were highly variable and substantially upregulated in IDCM. CYP26B1 was only detected marginally and not quantified. CYP26C1 was detected in human hearts, but levels did not differ significantly between donors and patients.

Given that resident cardiac ATRA levels are an indicator of heart health, we investigated the origins of low ATRA in HF. Steady-state ATRA is determined by a bevy of enzymes that influence its rate of biosynthesis from retinol, including the retinol dehydrogenases (RDHs), retinal reductases (e.g., DHRS3), and retinaldehyde dehydrogenases (RALDHs, e.g., ALDH1A1, ALDH1A2) (25, 26). Its rate of catabolism is largely determined by the CYP26 family of p450 hydroxylases (CYP26A1, CYP26B1, and CYP26C1) (26, 27). In the guinea pig proteome, Aldh1a1 (Figure 6A) declined 23% by 2 weeks (*n* = 3, *P* < 0.01, LIMMA-moderated *t* test) and 39% in HF (*n* = 3, *P* < 001). A second RALDH1-like protein and Aldh1a2 trended toward modest declines (12% and 15%) but were not significant (see Supplemental Figure 5). Finally, Aldh2, the broad specificity aldehyde dehydrogenase, though down early in guinea pig HF (see Supplemental Table 2), exhibited modest downregulation (16%, *n* = 3, *P* < 0.05). Based on enzyme levels as a first approximation, a decline in the rate of ATRA biosynthesis in guinea pig HF is initially consistent with reduced Aaldh1a1. CYP26s are poorly represented in trypsin-accessible proteomes owing to their low abundance, high isoelectric point, and membrane association. Transcript data (Figure 6A) indicated that neither Cyp26b1 nor Cyp26c1 mRNA levels changed in guinea pig HF. Cyp26a1 transcript levels were initially unchanged at 2 weeks, but declined significantly by 4 weeks (32%, *P* < 0.05), perhaps as a compensatory response to limit cellular ATRA decline.

Although IDCM shared low ATRA with guinea pig HF, levels of ATRA-metabolizing enzymes differed dramatically. Immunoblot analysis indicated that levels of ALDH1A1 (normalized to GAPDH) were several-fold higher in IDCM (Figure 6B). Among the CYP26s, CYP26B1 was only marginally detectable. CYP26C1 levels did not differ between the donor and patient groups. CYP26A1 levels were intriguing because they were highly variable, particularly in donor hearts. Levels were considerably higher in most patient samples. The near tripling of CYP26A1 levels was significant (*P* < 0.05).

### Targeted inhibition of ATRA catabolism limits cardiomyocyte hypertrophy in vitro.

**Figure 7:**
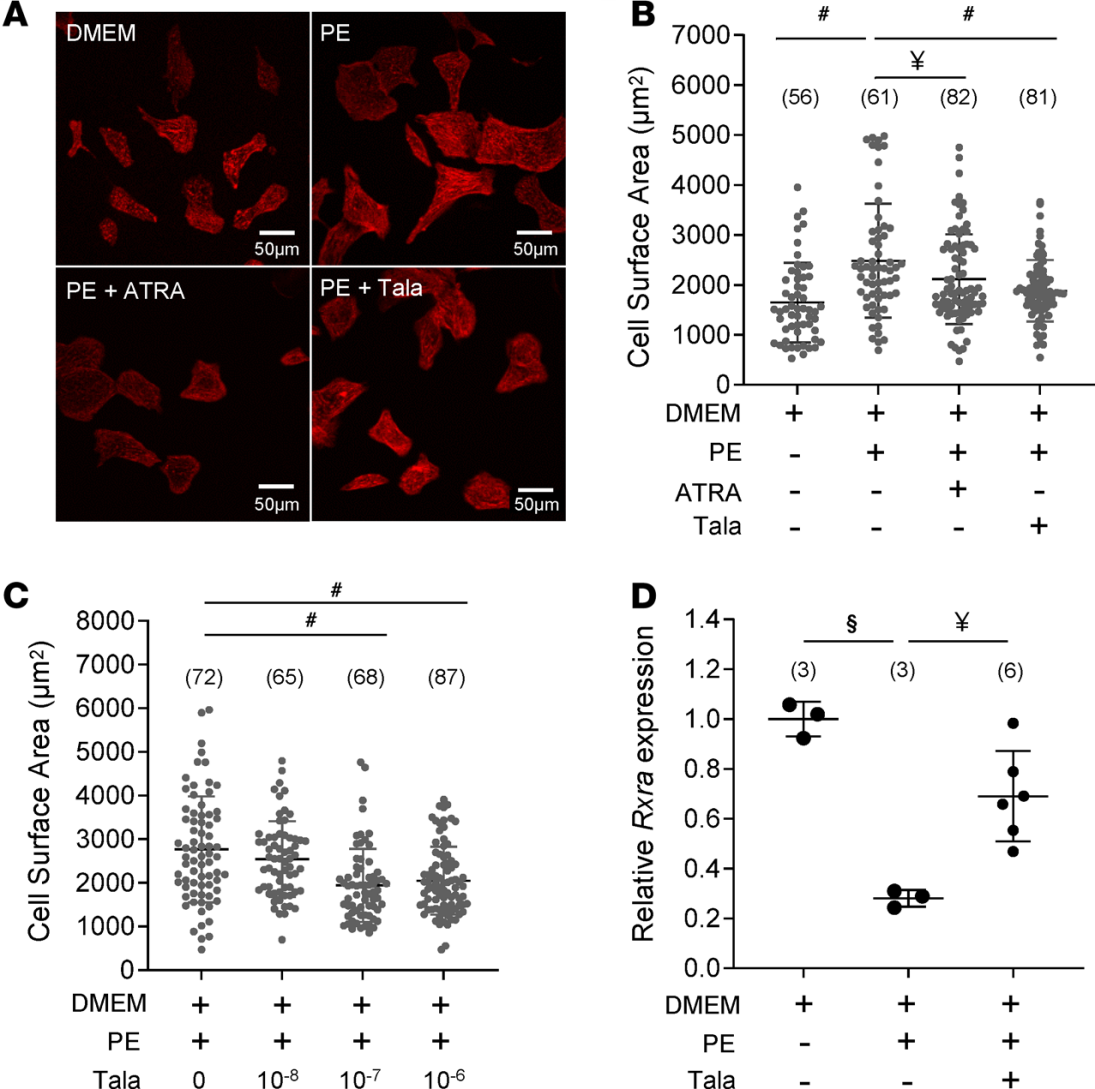
Inhibiting ATRA catabolism limits cardiomyocyte hypertrophy. (**A**) Images: NRVMs incubated in minimal medium (top left) in the presence of phenylephrine (PE; top right), PE plus ATRA (bottom left), or talarozole (Tala; bottom right). The scale is provided in the bottom right of the images. Data in **B**–**D** were analyzed by 1-way ANOVA with a post hoc Tukey’s HSD test. *N* values are given in parentheses. (^#^: *P* < 0.0001, ^§^: *P* < 0.001, ^¥^: *P* < 0.01). (**B**) Ensemble NRVM cross-sectional areas across the experimental groups are shown. ATRA (1 μM) prevented PE-induced cell enlargement. The CYP26 inhibitor Tala (1 μM) recapitulated mitigation of hypertrophy. (**C**) Concentration-dependent suppression of NRVM hypertrophy by Tala; 100 nM was sufficient to achieve maximum suppression. (**D**) Transcript levels of *Rxra* (an ATRA-responsive gene) in NRVMs were measured (by qPCR) as a proxy for levels of intracellular ATRA. PE decreased *Rxra* expression relative to control levels, but the decline was mitigated by coadministration of 1 μM Tala.

High cardiac CYP26A1 expression in human HF could, conceivably, compromise prospects for therapy predicated on ATRA supplementation, as it would likely limit the half-life of administered ATRA in situ. However, inhibition of CYP26 enzymes could limit the rate of ATRA clearance and potentially boost endogenous ATRA. To test this concept, we examined the impact of the pan-CYP26 inhibitor, talarozole, on α-adrenergic hypertrophy in neonatal rat ventricular myocytes (NRVMs; Figure 7). In replicated experiments, phenylephrine (PE) treatment of serum-starved NRVMs increased cross-sectional area on the order of 45%–80% (Figure 7A). As shown in Figure 7B, PE induced an increase in the cross-sectional area of 48% with respect to cells incubated in DMEM alone. Cotreatment with 1 μM ATRA blunted the hypertrophy (23% increase relative to DMEM). Talarozole, likewise at 1 μM, also suppressed PE-induced hypertrophy relative to PE (12% increase relative to DMEM). Figure 7C illustrates the concentration dependence of talarozole. Cell treatment with 100 nM talarozole was sufficient to elicit maximum suppression of PE-induced hypertrophy. Finally, we sought to determine the impact of both the hypertrophic stimulus and concomitant CYP26 inhibition on levels of intracellular ATRA. Yields of ATRA from NRVMs proved sufficiently low as to preclude direct quantitation by mass spectrometry. Therefore, we used qPCR to quantify the expression of the ATRA-responsive gene *Rxra* as a proxy for ATRA. After 24 hours in culture, PE reduced *Rxra* mRNA levels by 72% (*n* = 3, *P* < 0.001 relative to minimal media alone). Coadministration of talarozole with PE significantly limited the decline to 33% (*n* = 6, *P* < 0.01 relative to PE alone). Together, the results showed that boosting endogenous ATRA in NRVMs by inhibiting its catabolism was as effective as treatment with ATRA at limiting cardiomyocyte hypertrophy.

## Discussion

### Implication of ATRA decline in HF.

The potentially novel primary findings of this study are the following: (a) there was a resident cardiac ATRA decline in HF patients and a guinea pig model of HF, despite adequate tissue reserves of retinol; (b) remodeling of the retinoid metabolic program differed between human and guinea pig HF, with implications for a therapeutic strategy based on normalizing cardiac ATRA levels; and (c) inhibition of the CYP26 family of the cytochrome p450 hydroxylases with talarozole inhibited myocyte hypertrophy in vitro.

Rigorous isomer-specific quantification of retinoids is challenging, which may explain why the role of cardiac retinoid metabolism in the adult mammalian heart is understudied. Motivated by studies showing that low circulating retinoic acid is associated with poor cardiovascular outcomes and mortality (28), we sought to quantify the state of retinoids in failing human hearts. We showed that nonischemic, dilated, failing hearts exhibited a nearly 40% ATRA decline, even as retinol increased, consistent with an impairment in ATRA biosynthesis. Interestingly, ATRA has previously been shown to be elevated in a study of patients with severe HF (LVEF < 35%) and advanced coronary artery disease (29). The same study reported no difference in nonischemic failing hearts. Discrepancies may be attributable to differences in retinoid quantitation methodology, disease etiology, and comorbidities, among other factors. Nevertheless, what is clear is that aberrant intracardiac ATRA metabolism has been implicated in 2 etiologies of human HF. Notwithstanding that the HFrEF, HFpEF, IDCM, and ICM proteomes are at least consistent with low cardiac ATRA, systematic characterization of the retinoids across HF types and severities is warranted.

In this study, we also explicitly demonstrated an ATRA decline in a mammalian model of HF. The result is consistent with pharmacological studies showing that ATRA ameliorates maladaptive remodeling in a rat transverse aortic constriction model (30) and in the context of doxorubicin-induced cardiomyopathy (31, 32). Here, we showed that ATRA prevented HF and pulmonary congestion in a guinea pig model of HF and sudden cardiac death. How impaired ATRA homeostasis contributes to HF progression is poorly understood. Some clues may be garnered from in vitro retinoid signaling studies and mouse KO studies of one of ATRA’s cognate receptors, RAR-α. We showed, as others have (33, 34), that ATRA blunted α-adrenergic hypertrophic signaling in NRVMs (Figure 7). ATRA has been shown to upregulate MAPK phosphatases, Mkp1 and Mkp2, which inactivate Mek upstream of Erk1 and Erk2 (34). In mice, attenuating ATRA signaling by cardiomyocyte-specific KO of RAR-α leads to increased oxidative stress, altered calcium transients, and diastolic dysfunction (19). The oxidative stress was notable, as the ratio of reduced to oxidized cellular glutathione (GSH/GSSG) declined substantially (approximately 40%–50%), owing to the upregulation of ROS-generating enzymes like NADPH oxidase 2 and 4 (Nox2 and Nox4), and downregulation of ROS scavenging enzymes, SOD1 and SOD2. Altered Ca^2+^ transients in RAR-α conditional KO (cKO) hearts were characterized by slowed Ca^2+^ reuptake to the SR, which was associated with decreased expression of SERCA2a and decreased phosphorylation of phospholamban.

Our findings showed that interstitial fibrosis increased in the guinea pig HF model and that the extent was limited by ATRA treatment (Figure 5G). Levels of fibrosis were not altered in the cardiac RAR-α cKO model (19), in which RAR-α was specifically depleted in cardiomyocytes. This would suggest that attenuation of cardiomyocyte RAR signaling is insufficient to drastically alter local profibrotic myocyte signaling to neighboring fibroblasts. Reduction of fibrosis by ATRA in the guinea pig model is then likely due to direct RAR signaling in cardiac fibroblasts. This would be consistent with evidence that ATRA attenuates the growth of neonatal cardiac fibroblasts in vitro, the secretion of type I and III collagens, and TGF-β expression in response to angiotensin II (35). Taken together, the benefits of ATRA treatment in the guinea pig HF model likely stem from activating, or at least offsetting the decline of, protective signaling pathways across cell types of the heart.

ATRA signaling can be modulated either through changes in the effective cellular concentrations of ATRA or through altered levels and/or the intrinsic activation status of the RARs. Zhu et al. (19) showed that models of metabolic stress, such as streptozotocin-induced type I diabetes or a high-fat diet, alter the expression of RAR-α. In contrast, another study found no significant changes in the mRNA of RARs in guinea pig HF (21). We suggest that altered cardiac RAR signaling in HF stems, at least in part, from changes to the local availability of its ligand, ATRA.

### Changes in retinoid metabolism in guinea pig HF.

From Figures 4 and 6, we conclude that low cardiac ATRA levels in guinea pig HF did not stem from a deficit of the parent substrate, retinol. Rather, impaired retinol metabolism, either at the level of retinol conversion to retinaldehyde (RAL) by the RDHs, or RAL to ATRA by the RALDHs, could lead to low ATRA. RDHs (Rdh5, Rdh13, Rdh14) were detected in the guinea pig HF proteome but were not significantly regulated. One of the best-characterized RDHs, Rdh10, was not detected. However, since our iTRAQ proteomics workflow employed data-dependent peptide acquisition, the absence of evidence for Rdh10 should not necessarily be interpreted as evidence of its absence. At the level of RALDHs, Raldh1 (Aldh1a1) but not Raldh2 (Aldh1a2) was progressively, substantially, and significantly downregulated in guinea pig HF (39% by 4 weeks). The data presented are initially consistent with the downregulation of Raldh1 contributing to a bottleneck in ATRA biosynthesis from RAL in the guinea pig. The role of Raldh1 in the heart has received little scrutiny, perhaps since its expression is weak and temporally restricted in mouse cardiac development relative to Raldh2 (36), and Raldh2 polymorphisms have been associated with both congenital heart disease (37) and uncontrolled blood pressure (38). Nevertheless, our study showed that Raldh1 was dynamically regulated, not only in guinea pig but also in human HF. It bears noting, however, that further factors may contribute to the altered rates of ATRA biosynthesis in HF, including the levels and intracellular location of cellular retinol-binding proteins and cellular retinoic acid–binding proteins (CRABPs) (25), as well as the activities of the retinaldehyde reductases that limit the retinaldehyde pool. Targeted mass spectrometry assays, currently in development, will afford comprehensive identification and absolute quantification of the core retinoid-metabolizing enzymes of the heart.

### Divergent regulation of retinoid-metabolizing enzymes between human and guinea pig HF.

It is clear that despite a common metabolic endpoint (i.e., low ATRA), control of the retinoid metabolic network differs substantially between humans and guinea pigs. The data in Figure 6 offer a striking contrast in the levels of ATRA-synthesizing RALDHs and ATRA-catabolizing CYP26s between human HF patients and guinea pigs. As with the guinea pig HF, RALDH1 levels were dynamically regulated in human IDCM. Counterintuitively though, levels increased in human HF, as did levels of CYP26A1. Notwithstanding that the results shown in Figure 2 (i.e., decreased ATRA, increased retinol) are consistent with impaired ATRA biosynthesis, high CYP26A1 could also contribute to decreased cardiac ATRA levels in patients by accelerating its catabolism. The roles of CYP26s in adult physiology, let alone cardiac physiology, are poorly understood, but recent work with conditional global CYP26A1-KO mice indicates that this high-V_max_ form of CYP26 plays a key role in hepatic retinol homeostasis and clearance of exogenous ATRA (39). CYP26B1 is thought to have a greater role in extrahepatic tissues. Indeed, CYP26B1 has been shown to regulate ATRA metabolism in human aortic smooth muscle cells (40) and macrophages within atherosclerotic lesions (41). Moreover, a hyperactive CYP26B1 polymorphism (rs2241057, Leu264Ser) may aggravate atherosclerosis (41). However, Figure 6 clearly shows that cardiac CYP26A1 was dynamically regulated in both human and experimental HF, albeit fluctuating in opposite directions. It is unclear whether the divergent regulation of both RALDH1 and CYP26A1 reflects a genuine species difference in the transcriptional regulation of these enzymes or whether the etiology of HF may be a factor, since the guinea pig model of HF was elicited by pressure overload, whereas IDCM individuals had no overt hypertension. Moreover, the impact of the profound changes to neurohumoral signaling in end-stage human HF on ATRA metabolism is unknown, as is the impact of comorbidities. Again, deep profiling of the core ATRA metabolic program by mass spectrometry across classes and stages of HF should prove informative.

### Broader changes to retinoid signaling in HF.

Proteomic analysis of both human and guinea pig hearts (Figures 1 and 3) implicated RXR-dependent pathways in addition to ATRA/RAR-dependent pathways. RXRs are transcriptional regulatory proteins that interact with several binding partners including RARs, vitamin D receptors, glucocorticoid receptors, thyroid hormone receptors, and peroxisome proliferator–activated receptors, among others (42). RXRs, particularly RXR-α, are known to regulate the levels of ATRA-metabolizing enzymes. Specifically, CRABP2, RALDH1, RALDH2, and CYP26A1 have all been shown to be dependent on RXR transcriptional activity (43–47). However, given the binding promiscuity of RXRs, impaired RXR signaling likely has roles in HF progression that extend beyond a role in cardiac ATRA homeostasis. Prior work has shown that RXR-α dysregulation has been observed later in the pathogenesis of HF. Specifically, low transcript and protein levels have been shown to correlate with impaired fatty acid metabolism in tachy-paced dog hearts (48). This would be consistent with RXR-α’s role, complexed to PPARα, in transcriptional control of enzymes that catalyze fatty acid β-oxidation (e.g., medium-chain acyl-CoA dehydrogenase) (49–52).

In vitro, 9-*cis*-RA but not ATRA serves as a high-affinity ligand for RXRs that dramatically increases transcriptional activation of RXR target genes. However, it bears noting that we did not identify 9-*cis*-RA in either human or guinea pig hearts. This is consistent with prior retinoid MS quantification in rats, mice, and nonhuman primates, which showed that the only organ currently known to synthesize detectable levels of 9-*cis*-RA is the pancreas (53–55). This suggests that the involvement of the cardiac RXRs, in any stage of HF progression, may be subordinate to control by RXR-binding partners and their ligands including RARs/ATRA, the PPARs/essential fatty acids, and others.

### Challenges facing the development of retinoid therapy for HF.

As a potent hormone, ATRA levels are finely tuned physiologically, and establishing a therapeutic window could pose a challenge. ATRA has been used successfully to treat acute promyelocytic leukemia (APL) (56) and is generally well tolerated. About 10%–15% of patients experience a condition formerly known as retinoic acid syndrome (57), characterized by cardiorespiratory distress similar to acute respiratory distress syndrome, displaying fever, dyspnea, pleural and pericardial effusion, hypotension, and occasionally renal failure. But the syndrome has since been renamed APL differentiation syndrome, as it can also be triggered by treatment with arsenic trioxide, and thus is now believed to reflect the consequence of APL treatment rather than ATRA-specific toxicity at the commonly used dose (45 mg/m^2^).

Nevertheless, in studies focused on the hearts of healthy rats, 10 mg/kg/d, although ATRA did not adversely affect cardiac morphology or function at 2 months, it did elicit significant changes in cardiomyocyte cross-sectional area, as well as changes in indices of oxidative stress (58). Clearly, further animal model studies are required to determine the minimum effective ATRA dose to ameliorate HF.

Although retinoid quantitation results (Figures 2 and 4) are consistent with an impairment in ATRA biosynthesis in both human and guinea pig HF, in Figure 6, we also show high levels of CYP26A1 in IDCM patients. High levels of CYP26A1 enzyme would constitute a reserve of ATRA-clearing activity that could limit the impact of ATRA supplementation therapy. This scenario, of muted ATRA efficacy due to high CYP26 activity, has also been documented in APL (59) and has prompted the development of a class of CYP26 inhibitors, also known as retinoic acid metabolism blocking agents (RAMBAs), that have the potential to prolong the half-life of pharmacologically administered ATRA when used as adjuvant therapy in refractory patients (60). Talarozole (R115866 or Rambazole) is a third-generation RAMBA that displays more than 300-fold greater selectivity for CYP26 over other related cytochrome p450 hydroxylases, such as CYP17 and CYP19 (61, 62).

Intriguingly, CYP26 inhibition is sufficient to boost endogenous ATRA levels without the need for pharmacologically administered ATRA. Specifically, in rats, talarozole was sufficient to boost endogenous ATRA in the serum and liver and to induce expression of ATRA-responsive genes (63). In this study, we showed that treatment of NRVMs with as little as 100 nM of talarozole recapitulated suppression of α-adrenergic hypertrophy seen in response to exogenous ATRA (Figure 7). We also confirmed 1 μM talarozole was sufficient to offset the PE-induced decline in *Rxra* mRNA, which was chosen as a proxy for NRVM ATRA levels. The precise CYP26 gene targeted by talarozole in NRVMs is unclear because it is a pan-CYP26 inhibitor (63, 64). However, given that the IC_50_ for inhibition of hypertrophy is less than 100 nM and the IC_50_ for CYP26C1 inhibition is about 4 μM (64), talarozole likely acts through either CYP26A1 or CYP26B1.

### Conclusion.

ATRA is the latest example of a nuclear receptor–binding hormone whose levels are perturbed in human HF. Advanced HF in patients is associated with low serum levels of thyroid hormone (22, 65) and local cardiac hypothyroidism (23, 66, 67). The importance of intracardiac thyroid hormone levels was shown by cardiac-specific overexpression of type 2 triiodothyronine deiodinase, the primary enzyme responsible for the conversion of thyroid hormone to its active form, T3 (68). Although triiodothyronine deiodinase expression did not mitigate transverse aortic constriction–induced hypertrophy, it did prevent declines in contractility. Similar approaches, such as cardiac conditional overexpression of the RALDHs or KO of the CYP26s, will be key to defining the role of perturbed ATRA metabolism in HF.

## Methods

### Human myocardial tissue

Nonfailing hearts were obtained at the time of organ donation and failing human hearts were procured at the time of heart transplantation. Nonfailing donor hearts had an LVEF of more than 50%. IDCM hearts had an LVEF of 20% or less and dilated left ventricles. Hearts were arrested in situ using ice-cold cardioplegia solution, transported to the lab on wet ice, and flash-frozen within 4 hours of collection. Transmural myocardial samples were dissected from the mid-left ventricular free wall.

### Guinea pig HF/SCD model and ATRA treatment

Male Hartley guinea pigs (300 g) were obtained from Hilltop Lab Animals Inc. The guinea pig model of HF and sudden cardiac death (SCD) has been described previously (24). Briefly, the HF and SCD guinea pig model was produced by combining ascending aortic constriction and daily (nonhypertrophic) isoproterenol challenge (ACi model). As characterized previously (24), cardiac function of ACi animals is well compensated in the first 2 weeks (HYP) but declines rapidly thereafter (HF). Hypertrophic hearts were collected between 1 and 2 weeks after surgery (designated “2wk” in the figures), whereas failing hearts were collected at 4 weeks (“4wk”). After retrograde perfusion with 20 mL Tyrode’s solution, excised hearts were snap-frozen in liquid N_2_ and stored at –80°C. The treatment group consisted of guinea pigs subjected to the ACi protocols with the surgical implantation of an osmotic pump, which administered ATRA solubilized in palm oil and DMSO at a dose of 2 mg/kg/d.

### Echocardiography

Transthoracic echocardiography was performed on conscious guinea pigs at 4 weeks after surgery using a Vevo 2100 high-resolution in vivo imaging system with a 24 MHz transducer (VisualSonics). Two-dimensionally directed M-mode images were obtained from the short-axis views. Echocardiographic measurements were made on 3 consecutive cardiac cycles by the leading edge-to-leading edge method. Left ventricular end-diastolic and end-systolic dimensions and left ventricular end-diastolic posterior wall thickness were measured from the M-mode images, and left ventricular fractional shortening was calculated with the software VisualSonics v1.3.8.

### Histology

Levels of cardiac interstitial fibrosis were assessed by Masson’s trichrome staining. Briefly, hearts were excised and rapidly immersed in ice-cold saline solution, then fixed with 4% paraformaldehyde overnight. Tissues were then embedded in paraffin and sectioned into 5 μm slices along the short axis of the heart. Five slices were collected from the midventricular region of each heart and stained with Masson’s trichrome. Four regions from each slide were used to analyze interstitial collagen by computer-assisted image analysis (ImageJ, NIH). The data from the 4 regions were averaged to obtain the mean fibrosis level for each heart. Data were calculated from at least 3 biological replicates (*n* = 3 for Ctrl, *n* = 4 for 4-week HF and 4-week + ATRA).

### Quantification of retinoids

Vitamin A metabolites were quantified in a blinded manner. All samples were frozen at collection and stored at –80°C until extraction. Heart tissues were homogenized in saline and subjected to a 2-step liquid-liquid extraction under yellow lights as described previously (55, 69, 70). Internal standards were 4,4-dimethyl-RA and retinyl acetate. Levels of retinoic acid were determined by LC-MRM^3^ on a Shimadzu Prominence UFLC XR liquid chromatography system coupled to an AB Sciex 5500 QTRAP hybrid triple-quadrupole mass spectrometer using atmospheric pressure chemical ionization operated in positive ion mode as previously described (55). Retinol and retinyl esters were quantified via HPLC-UV as before (69). Retinoic acid, retinol, and total retinyl ester were normalized per gram of tissue. Statistical analysis of multigroup studies was performed by ANOVA with a post hoc Tukey’s honest significant difference (HSD) test. Two-group comparisons were performed using a Student’s 2-tailed *t* test.

### Analysis of proteomic and microarray data sets

#### Data summarization and statistics.

A proteomic study of cardiac tissue from 34 human subjects was published and is publicly available from the proteomeXchange database (PXD008934) (20). Proteins were originally normalized and quantified based on label-free quantitation (LFQ) ion intensities using MaxQuant (71). The data set was filtered to analyze proteins identified and quantified in at least 50% of patients. Missing data were interpolated using the *k* nearest-neighbors algorithm, and the data were transformed (log_2_) prior to performing empirical-Bayesian statistical analysis using a LIMMA multigroup comparison (72, 73), as implemented in Qlucore Omics Explorer 3.5 (Qlucore). Proteins were deemed differentially regulated if *P* was less than 0.05 (FDR = 12%).

The guinea pig cardiac proteome data set, available from proteomeXchange (PXD003980), was acquired using a peptide labeling MS^2^-based workflow (iTRAQ) and protein levels were determined by the “median sweep” algorithm for data normalization and protein summarization (21, 73). Missing data interpolation and statistical analysis were conducted as described for the human data set. At *P* less than 0.05, the FDR was 22%.

Select guinea pig cardiac mRNA levels were extracted from our guinea pig microarray data set (21) (e.g., Figure 6A) deposited in NCBI’s Gene Expression Omnibus (GEO), accessible through GEO series accession number GSE78077. Data were originally normalized and summarized using robust multiarray averaging and analyzed by 1-way ANOVA with a post hoc Tukey’s test for pairwise comparisons.

#### Dimension reduction, clustering, and expression profile ranking.

Features of the human HF proteomes were summarized in 2 dimensions using t-SNE (74) and hierarchical clustering, as implemented in Qlucore Omics Explorer 3.5 (Qlucore), filtered on differentially regulated proteins (*P* < 0.05 by LIMMA). Specifically, individual protein abundances were log-transformed and normalized across samples (mean = 0, var = 1) before agglomerative hierarchical clustering using Euclidean distance and complete linkage.

Differentially regulated proteins from the guinea pig HF data set were subjected to profile correlation ranking in Spotfire Decision Site with Functional Genomics 9.1.2 (TIBCO Spotfire) to determine which proteins most closely matched models of early, progressive, and late regulation. Specifically, group averages of each protein’s relative abundance were normalized by *z* scoring and compared with the models. Membership in each expression trajectory class was confined to proteins whose correlation with the model was greater than 0.975 (Pearson’s *r*).

#### Pathway and upstream regulator analysis.

Select protein clusters (human proteome) and kinetic profiles (guinea pig HF) were subjected to Ingenuity Pathway Analysis (QIAGEN). The Ingenuity knowledge base was used as the reference data set for pathway overrepresentation, which was assessed by Fisher’s exact test. Multihypothesis testing was addressed by reporting Benjamini-Hochberg corrected *P* values. Upstream Regulator Analysis (URA) was used to infer potential transcriptional regulators that would be consistent with coordinate protein regulation (Fisher’s exact test).

#### Construction of the functional protein association network.

Functional protein association/interaction networks were constructed by loading the UniProtKB identifiers of proteins downregulated in cluster 3 into StringApp 1.4.2 (75), embedded in Cytoscape 3.7.1 (76), and then searching the STRING v11 database (77). The default association/interaction threshold (STRING score > 0.4) was used to map relationships between proteins. Network modularity was assessed with the Markov clustering function in the ClusterMaker2 1.3.1 app (78) using the STRING score (>0.6) for edge weighting. The granularity parameter (inflation value) was set empirically. The final network is presented in an edge-weighted, spring-embedded layout using the Markov cluster (module) number for edge weighting, with modules arranged for maximum clarity.

### Culture of NRVMs

NRVMs were enzymatically dissociated from the ventricles of 2-day-old rats with trypsin. Freshly isolated NRVMs were resuspended in DMEM culture medium supplemented with 10% FBS, glucose, and vitamin B12. Two preplating steps were performed to enrich cardiac myocyte content in the culture. The final cell suspension was collected and plated at the desired density for the downstream experiment. For RT-PCR application, 1 × 10^6^ cells/well were plated in 6-well plates coated with 0.5% gelatin. For microscopy applications, 1.5 × 10^5^ cells/plate were seeded in 35 mm glass-bottom culture dishes coated with 0.5% gelatin. After 24-hour initial attachment in 10% FBS-containing media, cells were kept in serum-free media containing DMEM with 1% penicillin/streptomycin and 0.1% insulin-transferrin-selenium-X. After 12 hours of serum starvation, drug treatments were initiated. Experiments were terminated after 48 hours of drug treatment.

### Measurement of NRVM hypertrophy

Cells plated on 35 mm glass-bottom microscopy dishes were washed 3 times with prewarmed PBS. The cells were fixed in 4.7% paraformaldehyde in PBS for 10 minutes, after which cells were washed an additional 3 times in warmed PBS. The fixed cells were permeabilized in a solution containing 0.1% Triton X-100 in PBS for 4 minutes and washed with PBS. Cells were blocked in solution containing 1% BSA in PBS for 25 minutes, then stained with Alexa Fluor 594 phalloidin (Thermo Fisher Scientific) for 25 minutes and washed 3 times in PBS. Cells were imaged with an Andor Revolution X1 spinning disk confocal inverted microscope at 40× magnification. Cell surface area was measured by ImageJ (NIH). Specifically, images were converted to 8-bit and contrast-enhanced by 1%. Grayscale images were made binary using the mean auto local threshold. Cell area was determined with the FIJI analyze particles function to detect cells larger than 100 μm^2^, excluding cells on the edge of the image. The areas of individual cells were recorded from ROI Manager. Group area means were analyzed by 1-way ANOVA with a post hoc Tukey’s HSD test.

### Quantitation of RXR-α mRNA

Total RNA was extracted from NRVM cells with QIAGEN RNeasy spin columns. Transcripts were quantified using reverse-transcriptase quantitative PCR (RT-qPCR) on a Bio-Rad CFX384 system using 1-Step RNA to Ct TaqMan Master Mix (Thermo Fisher Scientific). The 10 μL RT-qPCR reactions were multiplexed to contain TaqMan assay Rn00441185 for rat RXR-α and Rn99999916 for Gapdh. The levels of RXR-α transcript were expressed relative to Gapdh using the 2^–ΔCt^ formula. Mean transcript levels were analyzed by 1-way ANOVA with post hoc Tukey’s HSD test.

### Immunoblot analysis

Protein concentrations of human cardiac tissue were determined using a Bio-Rad DC assay. From each heart, 20 μg protein was resolved by 4%–12% Bis-Tris gel electrophoresis (NuPAGE, Invitrogen). Proteins were blotted using the Bio-Rad TransBlot Turbo apparatus (7 minutes at 2.5 A). Membranes were blocked for 1 hour (5% [w/v] nonfat milk in Tris-buffered saline with 1% [w/v] Tween 20) and incubated overnight with primary antibodies in blocking solution at 4°C. The following antibodies were used: anti-CYP26A1 (Abcam, ab172474, 1/1000), anti-CYP26B1 (Thermo Fisher Scientific, PA5-15214, 1/1000), anti-CYP26C1 (Abcam, ab80226, 1/100) anti-ALDH1A1 (Abcam, ab52492, 1/1000), and anti-GAPDH (Cell Signaling Technology, 14C10, 1/2000). Membranes were washed and incubated with anti-rabbit antibody conjugated to horseradish peroxidase (MilliporeSigma, A0545, 1/80,000) antibodies for 1.5 hours at room temperature and probed for peroxidase activity using SuperSignal West Pico or West Femto Maximum Sensitivity Chemiluminescent Substrate. Films were digitized (.TIF), converted to 8-bit grayscale in ImageJ (v1.52a, NIH) and background was subtracted (rolling ball radius: 50). Immunoblot signal intensities of the CYP26 and ALDH1A1 blots were quantified and normalized to GAPDH intensities. Mean intensities for donors and IDCM patient groups were compared by 2-tailed Student’s *t* test.

### Statistics

Statistical analysis was conducted as described in each method above and in the figure legends. Error bars in dot plot graphs represent the standard deviation about the mean. Results were considered statistically significant if *P* was less than 0.05, irrespective of the test used.

### Study approval

The procurement of deidentified human myocardial tissue was performed under IRB protocols approved at the University of Pennsylvania (Philadelphia, Pennsylvania, USA) and Johns Hopkins University (Baltimore, Maryland, USA) as previously described (20, 79). Guinea pigs were housed in an animal care facility at the Johns Hopkins University School of Medicine, in conformance with the *Guide for the Care and Use of Laboratory Animals* published by the NIH (publication no. 85-23, revised 1996, National Academies Press), with the approval of the Johns Hopkins University Animal Care and Use Committee.

## Author contributions

NY, LEP, BOR, MAK, and DBF designed the experiments. KBM provided human heart tissues and data. NY, LP, JY, JWJ, and TL performed the experiments. NY, LEP, BOR, MAK, and DBF analyzed the data. DBF wrote the manuscript. NY, LEP, TL, KNP, CCT, KBM, BOR, and MAK edited the manuscript.

## Supplementary Material

Supplemental data

Supplemental Table 1

Supplemental Table 2

## Figures and Tables

**Table 1:**
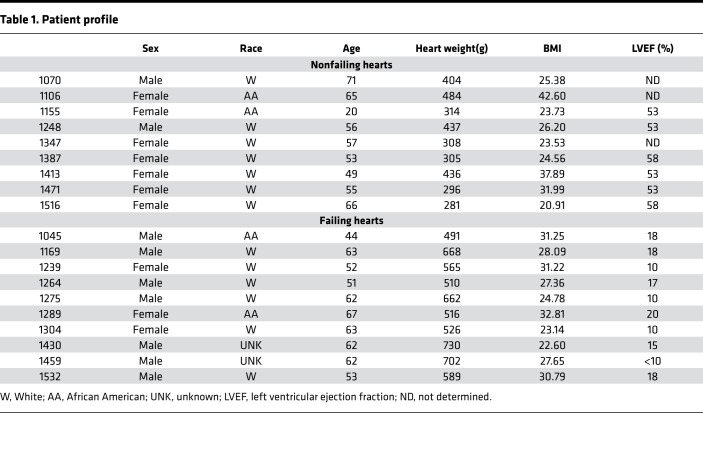
Patient profile

## Version 1 03/16/2021

In-Press Preview

## Version 2 04/22/2021

Electronic publication
