# Supplementary material for: Cardiac retinoic acid levels decline in heart failure
Source: JCI Insight. 2021 Apr 22;6(8):e137593. doi: 10.1172/jci.insight.137593 (PMC8119182; doi:10.1172/jci.insight.137593)
Supplement: Supplemental data [file jciinsight-6-137593-s073.pdf]

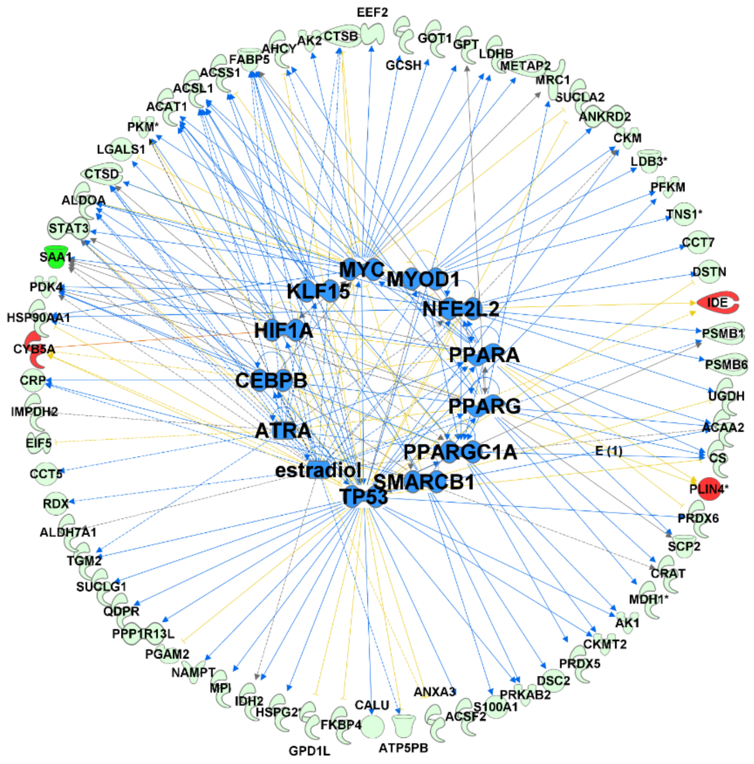

Figure S1. Network representation of Upstream Regulator Analysis. Top putative upstream transcriptional regulators (hub) from figure 1E are shown mapped to 60 proteins in cluster 3 for which there is a documented relationship (periphery). green = downregulated protein, red = upregulated protein, blue = inferred inhibition, yellow = relationship is at odds with prediction. 10 of the proteins in cluster 3 have been documented as ATRA-responsive. ATRA is also known to regulate activity or expression of 9 of the 11 TFs in the network hub and therefore could influence expression levels of proteins in cluster 3.

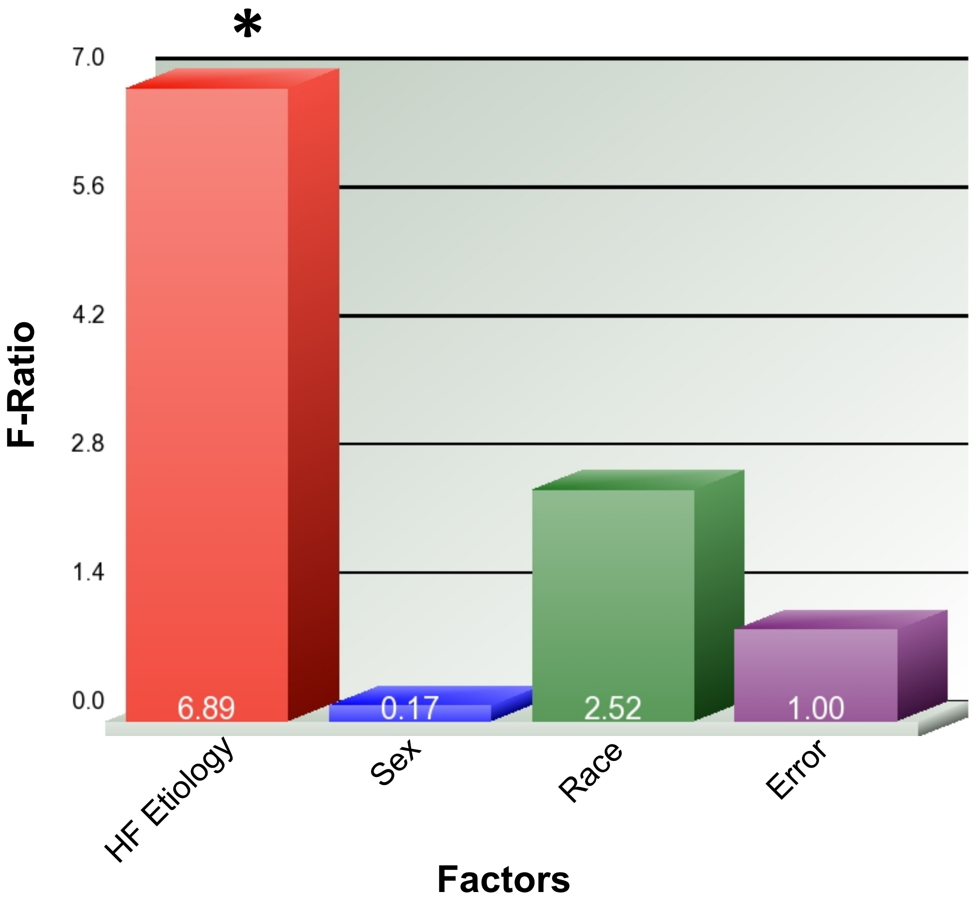

Figure S2. Contribution of Factors to ATRA variance. ATRA levels human myectomy samples were analyzed by 3-Way ANOVA to determine which factors, namely, HF diagnosis, sex, or race contributed to ATRA variance (F ratio = between group variability/within group variability). HF diagnosis contributed most to inter-group variance ($F=6.89$, $p=0.019$). Neither race nor sex contributed significantly.

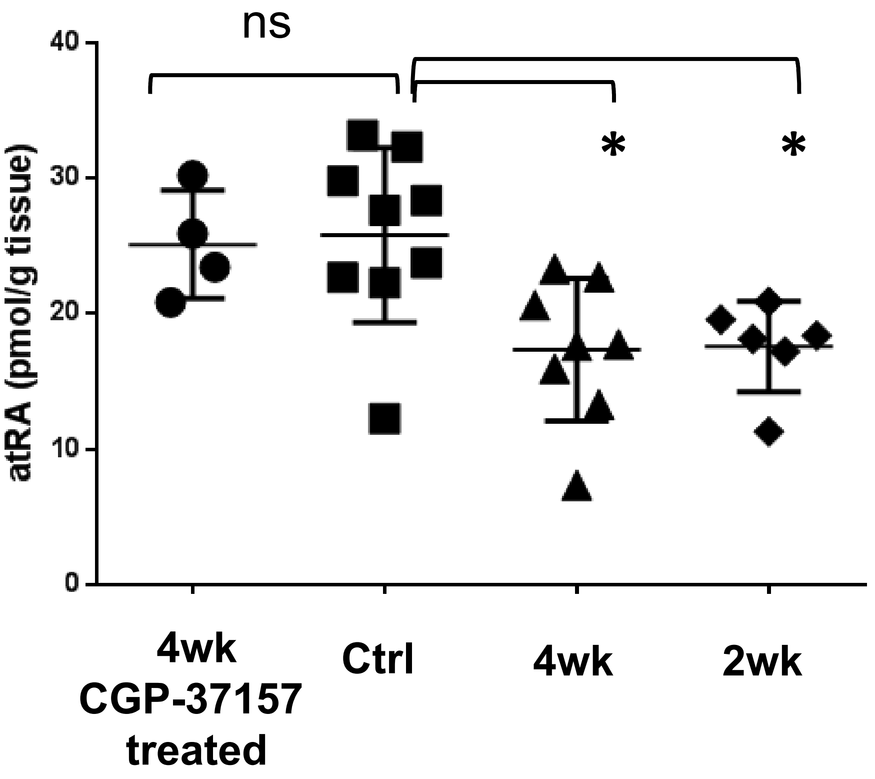

Figure S3. Cardiac ATRA deficit is prevented at 4wks of ACi protocol by cardioprotective treatment with CGP37-157 (ns: not significant at p<0.05).

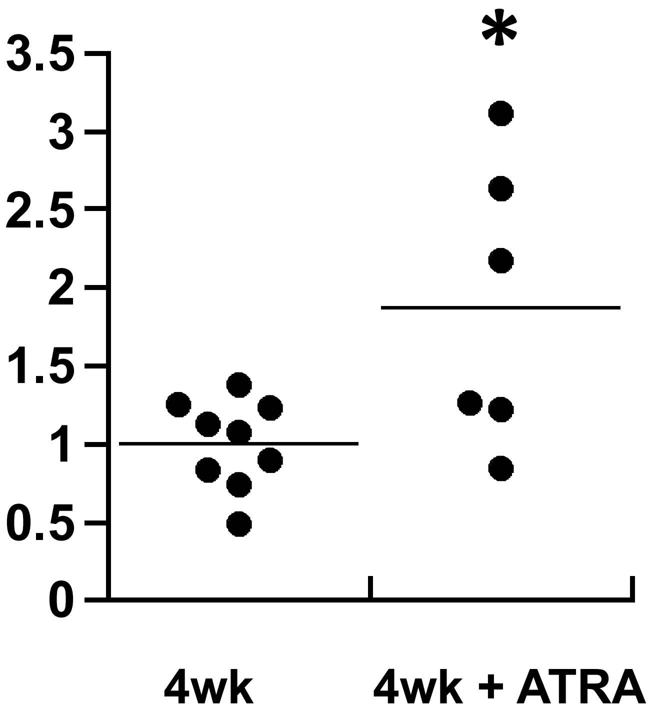

Figure S4. ATRA treatment of guinea pigs subjected to the 4wk aortic constriction protocol raises levels of intracardiac ATRA.

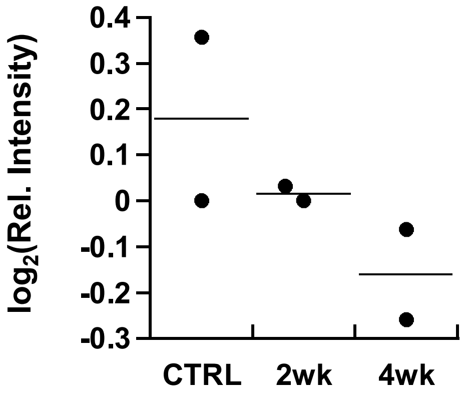

GI Accession: 348573107

retinal dehydrogenase 1-like [Cavia porcellus]

n=2, not significant at $p<0.05$

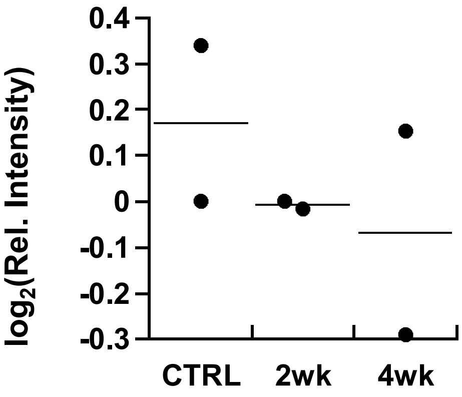

GI Accession:348552582

retinal dehydrogenase 2-like [Cavia porcellus]

n=2, not significant at $p<0.05$

Fig. S5. Other retinaldehyde dehydrogenase forms detected in the guinea pig proteome. Each trended toward modest downregulation though neither met the significance threshold ($p<0.05$ by LIMMA).
