# Supplementary material for: Cardiac retinoic acid levels decline in heart failure
Source: JCI Insight. 2021 Apr 22;6(8):e137593. doi: 10.1172/jci.insight.137593 (PMC8119182; doi:10.1172/jci.insight.137593)
Supplement: Supplemental Table 1 [file jciinsight-6-137593-s074.doc]

|  | **CTRL** | **4wk HF** | **4wk + ATRA** |
| --- | --- | --- | --- |
| **Heart Weight (HW; g)** | 1.82 (n=7; S.D.=0.23) | 2.78 (n=7; S.D.=0.13) | 2.12 (n=6; S.D.=0.34) |
| **Lung Weight (LW; g)** | 2.57 (n=7; S.D.=0.17) | 4.27 (n=7; S.D.=1.58) | 2.50 (n=6; S.D.=0.40) |
| **Tibia length (TL)** | 38.9 (n=7; S.D.=2.08) | 37.8 (n=7; S.D.=0.93) | 37.3 (n=6; S.D.=0.40) |
| **HW/TL** | 0.46 (n=7; S.D.=0.06) | 0.73 (n=7; S.D.=0.04) | 0.56 (n=6; S.D.=0.08) |
| **LW/TL** | 0.66 (n=7; S.D.=0.04) | 1.13 (n=7; S.D.=0.41) | 0.67 (n=6; S.D.=0.10) |
| **Fractional Shortening (%)** | 37.9 (n=6; S.D.=2.07) | 26.5 (n=8; S.D.=6.55) | 37.4 (n=10; S.D.=5.37) |
| **Ejection Fraction (%)** | 65.0 (n=6; S.D.=3.14) | 48.9 (n=8; S.D.=10.1) | 64.7 (n=10; S.D.=7.03) |
| **Heart Rate** | 312.5 (n=6; S.D.=30.9) | 314.4 (n=8; S.D.=30.6) | 274.5 (n=10; S.D.=33.9) |
| **LVID_d_ (cm)^a^** | 0.91 (n=6; S.D.=0.06) | 0.99 (n=8; S.D.=0.05) | 0.96 (n=7; S.D.=0.08) |
| **LVID_s_ (cm)^b^** | 0.56 (n=6; S.D.=0.04) | 0.73 (n=8; S.D.=0.07) | 0.59 (n=7; S.D.=0.08) |
| **IVSD (mm)^c^** | 2.19 (n=6; S.D.=0.23) | 2.63 (n=8; S.D.=0.27) | 2.24 (n=7; S.D.=0.16) |
| **PWTED (mm)^d^** | 1.96 (n=6; S.D.=0.26) | 2.40 (n=8; S.D.=0.26) | 2.03 (n=7; S.D.=0.22) |
| **LV Vol_d_ (ul)^e^** | 451.9 (n=2; S.D.=15.7) | 538.5 (n=7; S.D.=44.58) | 523.1 (n=7; S.D.=99.8) |
| **LV Vol_s_ (ul)^f^** | 146.8 (n=2; S.D.=10.9) | 277.2 (n=7; S.D.=59.8) | 182.7 (n=7; S.D.=66.0) |

**Supplemental Table 1. Cardiac Morphometry including Echocardiography**

**^a^** Left ventricular internal dimension at end-diastole

**^b^** Left ventricular internal dimension at end-systole

**^c^** Intraventricular septum thickness at end-diastole

**^d^** Posterior wall thickness at end-diastole

**^e^** Left ventricular volume at end-diastole

**^f^** Left ventricular volume at end-systole
